# Re-record and Spore Ultrastructure of *Nosema melasomae* Sidor & Jodal 1986, a Microsporidian Pathogen of *Crysomela populi* (Coleoptera: Chrysomelidae)

**Published:** 2018

**Authors:** Mustafa YAMAN

**Affiliations:** 1. Dept. of Biology, Faculty of Science, Karadeniz Technical University, 61080, Trabzon, Turkey; 2. Faculty of Arts and Science, Abant İzzet Baysal University, 14030, Bolu, Turkey

**Keywords:** *Nosema melasomae*, Sporeultrastructure, Microsporidium, *Crysomela populi*, Chrysomelidae

## Abstract

**Background:**

*Chrysomela (=Melasoma) populi* is one of the most serious pests on poplar plantations. In the present study, a microsporidian pathogen, *Nosema melasomae* infecting *Crysomela populi* is re-recorded from a new geographical locality and its spore ultrastructure is given for the first time.

**Methods:**

Larvae and adults of *C. populi* were dissected in Ringer’s solution and prepared wet smears were examined under a microscope. Detected fresh and stained spores were measured and photographed using an Olympus BX51 microscope with a DP-25 digital camera and a DP2-BSW Soft Imaging System. The ultrastructure of the pathogen was studied with a Philips EM 208 transmission electron microscope using standard preparation techniques as previously described

**Results:**

Fresh spores of the microsporidian pathogen are elongate, 4.86 ± 0.71 μm in length and 1.64 ± 0.19 μm in width. The spore wall is considerable thin, measured 60 to 100 nm and consists of a clear endospore (40 to 80 nm) and an electron-dense, uniform exospore (15 to 30 nm). The polar filament is isofilar and has only 6–8 coils. Nuclei in the cell are 400–560 nm in diameter. The polaroplast has a thin lamellated structure.

**Conclusion:**

The pathogen from *C. populi* is *N. melasomae* Sidor & Jodal, 1986 and its systematic position given by Sidor and Jodal. The spore ultrastructure of *N. melasomae* differs from those of other microsporidia infecting chrysomelids.

## Introduction

The family Chrysomelidae (Coleoptera) includes over 35,000 species in more than 2,500 genera in the world, making it one of the largest and most important agricultural and forest pests. One of members of this family, *Chrysomela (=Melasoma) populi* is known as one of the most serious pests on poplar plantations (1).

Most common method to control this pest, chemical control was extensively used and it is known as the effective control strategy. However, in pest control strategies, biological control is the most favourable method for poplar pests. Unfortunately, there is no much knowledge concerning with biological control of *C. populi* or its natural enemies. Owing to a lack of information on other control strategies suppressing *C. populi* populations, studies of its natural enemies are of great importance for ecologically alternative control programs.

Chrysomelids are frequently infected by microsporidia (2–7). Most microsporidia in the family *Chrysomelidae* are known as extremely pathogenic, and they can parasitize a large proportion of the host population (8). The first microsporidian described from this family was *Nosema phyllotretae* Weiser 1961, found in *Phyllotreta atra.* Today, over 28 species of Microsporidia have been described from the family Chrysomelidae. Most of them infect only one host, however our knowledge show that some of them infect more than one host. On the other hand, most of previous descriptions are based on light microscopic observations like as that in *C. populi*. Therefore it becomes more important to study taxonomic characters and compare taxonomically microsporidia infecting chrysomelids for true descriptions.

Twenty-five years after the first microsporidian pathogen recorded from *P. atra,* a member of the family Chrysomelidae, Sidor and Jodal (9) identified *N. melasomae*, sole microsporidium from *Chrysomela* (*=Melasoma*) *populi.* Their description was based on light microscopy. Later Zeki and Toros (10) mentioned that they observed microsporidium pathogen in *C. populi* populations in Turkey without any description and micrographs. Since this record, no microsporidium causing infections in natural populations of *C. populi* has been recorded from *C. populi.*

In the present paper, the re-record and spore ultrastructure of *N. melasomae* in *C. populi* are presented, the ultrastructural characteristic features of the pathogen are given and compared with other *Nosema* species infecting beetles in the family Chrysomelidae (Coleoptera) for the first time.

## Material and Methods

### Insect collections

*C. populi* shows an extensive distribution in Turkey. Previously Yaman (11) searched the distribution of a neogregarine pathogen of this pest from thirteen localities in a wide broad geography in Turkey during the years 2013 and 2014. In this extensive study they did not observed any microsporidium infection in *C. populi* populations. Therefore we directed our study to new localities, Tokat, Çorum, Yozgat, Ankara, Kırşehir, Karabük in Turkey in July-2015, where there has been no any pathogen record from *C. populi* populations before.

### Light and electron microscopy

Overall, 149 beetles were dissected in Ringer’s solution and prepared wet smears were examined under a microscope for detection of microsporidian spores. The slides suspected with microspordian infections were air-dried, fixed with methanol for 2–3 min and then stained overnight freshly prepared 5% solution of Giemsa stain, and re-examined under the microscope for stained life stages of the pathogens. Detected fresh and stained spores were measured and photographed using an Olympus BX51 microscope with a DP-25 digital camera and a DP2-BSW Soft Imaging System.

The ultrastructure of the pathogen was studied with a Philips EM 208 transmission electron microscope using standard preparation techniques as previously described (2).

## Results

**Fig. 1–2:**
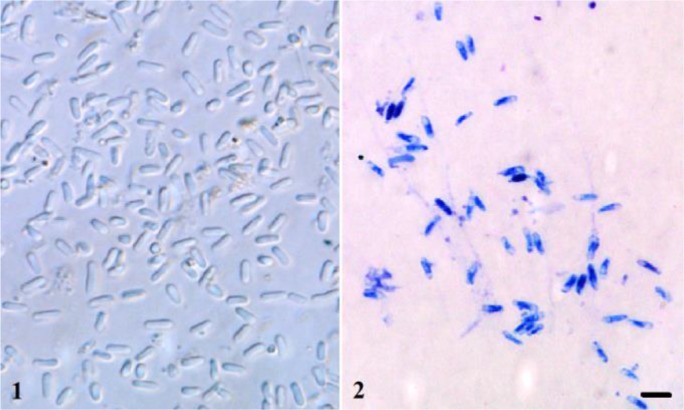
Spores of *Nosema melasomae* from *Crysomela populi* in fresh 1) and Giemsa-stained 2) smears. bar= 5 μm

During the study microsporidian infection was observed in one population in Kırşehir (Turkey). The infection was found in an adult. Only one (0.7%) of 149 beetles was infected by the microsporidian pathogen. Hemolymph and midgut was the infection site. Free spores in direct contact to the host cell cytoplasm were observed during the light and electron microscopically observations. Fresh spores of the microsporidian pathogen are elongate, 4.86 (3.43–6.78) ± 0.71 μm in length and 1.64 (1.30–2.03) ± 0.19 μm in width (Fig. 1). Giemsa-stained spores are 4.26 (3.19–5.60) ± 0.56 μm in length and 1.17 (0.77–1.54) ± 0.17 μm in width (Fig. 2).

**Fig. 3–7:**
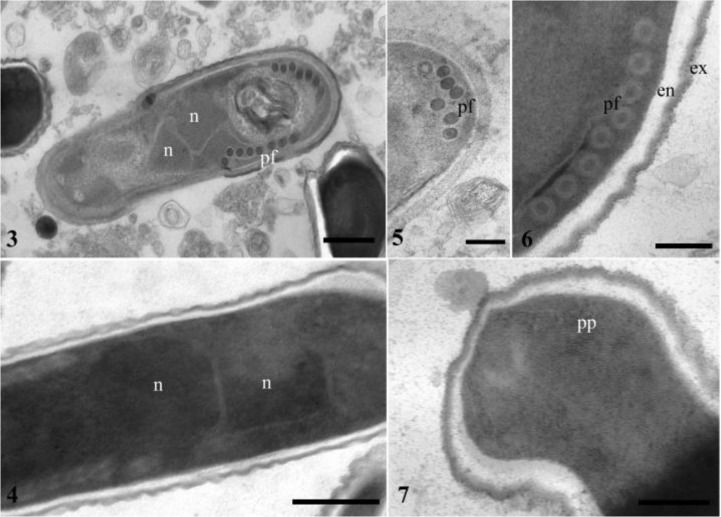
Ultrastructures of spores of *Nosema melasomae* in *Crysomela populi*. **3.** A diplokaryotic young spore with seven inmature polar filament coils. Bar = 1 μm **4**. Section of posterior part of a young spore with six isofilar polar filament coils (Note that exospore is not clearly seen). bar = 500 nm **5**. Section of posterior part of a mature spore with eight isofilar polar filament coils (pf) (Note that both thick endospore (en) and exospore (ex) is well seen). bar = 200 nm **6.** A clearly visible diplokaryon (n) in the longitudinal section of a mature spore bar = 150 nm **7**. Anterior part of a mature spore with thin lamellae polaroplast. bar = 500 nm

The elongate spores are diplokaryon (Fig. 3 and 4). Nuclei in the cell are 400–560 nm in diameter (Fig. 4). The spore wall is considerable thin, measured 60 to 100 nm and consists of a clear endospore (40 to 80 nm) and an electron-dense, uniform exospore (15 to 30 nm) (Fig. 4–6). The polar filament is seen as isofilar in both young and mature spores and has only 6–8 polar filament coils (Fig. 3, 5, 6). Diameter of the polar filament coils is 45–60 nm. The well-developed polaroplast has a lamellated structure with thin lamellae (Fig. 7).

## Discussion

*Nosema melasomae* Sidor & Jodal, 1986 is the sole microsporidian pathogen, firstly found and described by Sidor and Jodal (9) in *C. populi* and there has been no later record of any microsporidium species from this pest with one exception (10) without any description or micrograph. In the present study, re-record and spore ultrastructural characteristics of *N. melasomae* from *C. populi* are presented for the first time after its first description in the former Yugoslavia, which was based solely on light microscopy (9).

Up to now eleven *Nosema* species have been described from the members of the family Chrysomelidae (11). Their morphological characteristics, infection sites, host insects and localities are given in Table 1.

**Table 1:** *Nosema* species described in the family Chrysomelidae (Coleoptera)

***Nosema species***	***Spore size (μm)***	***Infected organ***	***Host***	***Locality***
*Nosema phyllotretae* Weiser, 1961 (20)	4.2 × 2–3	Adipose body	*Phyllotreta atra* *Phyllotreta undulate*	England
*Nosema phyllotretae* Yaman et al., 2005 (3)	4.08 × 2.53	General infestation	*Phyllotreta atra*	Turkey
*Nosema gastroideae* Hostounský and Weiser, 1973 (21)	3–4.8 × 2.5–3	Overall infestation	*Gastrophysa polygoni* and several experimental hosts	Czechoslovakia
*Nosema polygrammae* Hostounský and Weiser, 1975 (22)	4.8 × 2.05	Gut	*Polygramma undecemlineata*	Cuba
*Nosema equestris* Hostounský and Weiser, 1980 (23)	4–5 × 3	General infestation	*Gastrophysa viridula Leptinotarsa decemlineata*	Czechoslovakia
*Nosema couilloudi* Toguebaye and Marchand, 1984 (16)	3.4 –4 × 1–1.5	Gut	*Nisotra* sp.	Senegal
*Nosema birgii* Toguebaye and Marchand, 1986 (17)	6.2 × 3.5	Eggs and general infestation, larvae and imago	*Mesoplatys cincta*	Senegal
*Nosema nisotrae* Toguebaye and Marchand, 1989 (18)	5.8 × 3.1	General infestation	*Nisotra* sp.	Senegal
*Nosema galerucellae* Toguebaye and Bouix, 1989 (19)	4.95 × 2.89	Gut principally, adipose body, muscles, tracheae and Malpighian tubules	*Galerucella luteola*	France
*Nosema chaetocnemae* Yaman and Radek, 2003 (2)	3.52 × 2.09	Gut, tracheae, muscles and Malpighian tubules	*Chaetocnema tibialis*	Samsun, Turkey
*Nosema tokati* Yaman et al., 2008 (4)	3.82 × 1.3	Malpighian tubules	*Chaetocnema tibialis*	Tokat, Turkey
*Nosema leptinotarsae* Lipa, 1968 (24)	2–5 × 1.9–3.3	Haemolymph	*Leptinotarsa decemlineata*	U.S.S.R.
*Nosema leptinotarsae* Yaman et al., 2011 (6)	4.69 × 2.43	General infestation	*Leptinotarsa decemlineata*	Turkey
*Microsporidium* sp.*1* Yaman et al., 2015 (11)	3.66 to 5.66 × 1.35 to 2.22	Haemolymph	*Crepidodera aurata*	Turkey
*Microsporidium* sp.*2* Yaman et al., 2015 (11)	2.44 to 3.55 × 1.25 to 1.55	Haemolymph	*Crepidodera aurata*	Turkey
*Nosema melasomae* Sidor and Jodal, 1986 (9)	3.75–5.25 (4.17) × 1.86–2.62 (2.26)	Haemolymph, epithelium	*Cyrsomela populi*	Yugoslavia
*Nosema melasomae* The present study	4.86 × 1.64	Haemolymph, gut	*Cyrsomela populi*	Turkey

In morphological characteristics, spore dimension is a good taxonomic feature for comparison of species of microsporidia infecting similar group insects. As seen in Table 1, *N. melasomae* recorded from Turkey is characterized with the longer and narrower spore size. The spore size (4.86 μm) of the pathogen recorded from *C. populi* takes place in the length range (3.77–5.25 μm), but not (1.64 μm) in the wide range (1.86–2.62 μm) of the spore, given for *N. melasomae* (9). *N. melasomae* (4.86 μm in length) recorded from *C. populi* in Turkey is longer and narrower than the original described pathogen in the former Yugoslavia. *N. melasomae* from Turkey also differs from all other *Nosema* species in spore size, which were described from chrysomelid hosts.

Ultrastructural characters are used mostly in the classification of microsporidia (12). The spore is one of the most important life cycle stages and always present, and it is known as the main diagnostic element. In recent identification keys to microsporidium genera, ultrastructural characteristics of spores are always included (13) and good criteria so that provide abundant features to evaluate and compare microsporidia infecting similar host insects in the same family (3, 5, 14). In the literatures, spore ultrastructures belonging to eight of the twelve *Nosema* species recroded from Chrysomelid hosts have been studied. Ultrastructural characteristics of the eight *Nosema* species are given in Table 2. It is clearly seen that *N. melasomae* differs in a combination of three ultrastructural characteristics such as thickness of the spore wall, diameter of the polar filament and number of the polar filament coils. *N. melasomae* has considerable thin spore wall (60 to 100 nm) and narrow polar filament (45–60 nm in diameter). As seen in Table 2, it also shows difference in the number of the polar filament coils (6–8 coils). The number of polar coils is accepted as an important taxonomic criterion for differentiating species (15). *N. melasomae* has the lowest number of polar filament coils between the *Nosema* species infecting chrysomelids. The number of polar coils of *N. melasomae* (6–8) can show similarity with that of *N. galerucellae* (7–9 coils). However it clearly differs from *N. galerucellae* in the thickness of spore wall, spore size, infected host species and also locality of the host population (Table 1 and 2). As a result, compared to the other *Nosema* species infecting beetles in the family Chrysomelidae, *N. melasomae* has the thinnest spore wall, the narrowest polar filament and the lowest number of polar filament coils (6–8 coils).

**Table 2:** Some *Nosema* species described in the family Chrysomelidae (Coleoptera) and their morphological and ultrastructural features

***Nosema species***	***Host***	***Spore *measurements**	***Ultrastructural features***			***Reference***
			**Polaroplast**	**Spore wall (nm)**	**Polar filament**	
*Nosema couilloudi*	*Nisotra* sp.	3.4∼4 × 1∼1.5	Lamellar	60	8–10 coils	(16)
*Nosema birgii*	*Mesoplatys cincta*	6.2 × 3.5	Lamellar and vesicular	---	12–14 coils	(17)
*Nosema nisotrae*	*Nisotra* sp.	5.8 × 3.1	Tubular	65–155	15–18 coils	(18)
*Nosema galerucellae*	*Galerucella luteola*	4.95 × 2.89	Lamellar	80–100	7–9 coils	(19)
*Nosema chaetocnemae*	*Chaetocnema tibialis*	3.52 × 2.09	Relatively vesicular	176.5–213	13 coils	(2)
*Nosema phyllotretae*	*Phyllotreta atra*	4.08 × 2.53	Lamellar	110–175	13–15 coils	(3)
*Nosema tokati*	*Chaetocnema tibialis*	3.82 × 1.3	Lamellar	85–100	8–10 coils	(4)
*Nosema leptinotarsae*	*Leptinotarsa decemlineata*	4.69 × 2.43	Lamellar	180–250	15–16 coils	(6)
*Nosema melasomae*	*Cyrsomela populi*	4.86 ± 1.64	Lamellar	60–100	6–8 coils	Present study

During the study, only one adult sample was found to be infected between the examined 149 beetles. The total infection rate recorded in this study is very low (0.7%). In contrast, Sidor and Jodal (9) found *N. melasomae* infection in the larvae with 53.5 to 82% infection rates. During the study we also examined 23 living larvae from the same locality to search microsporidian infection, but there was no infection. It was difficult to find dead larvae on the young poplar trees; therefore, to search microsporidian infection in dead larvae was not possible. This can be reason of why the infection rate was found too low.

In microsporidian taxonomy, descriptions based mainly on light microscopic observations are not sufficient to true description and result in the unnecessary creation of new species. Especially, it is often difficult to compare microsporidian species infecting similar group insects in the same family because of that some microsporidia infects more than one host. Therefore ultrastructural details are always needed to identify pathogens and they provide useful information to discriminate microsporidia. In the present study, the findings confirm and justify the identification and classification of *N. melasomae* originally described from *C. populi* as a separate species (9), based on light microscopic observations. Furthermore the results provide useful information for the identification and comparison of other *Nosema* species from chrysomelid hosts.

## References

[1] Urban J Occurrence, bionomics and harmfulness of *Chrysomela populi* L. (Coleoptera; Chrysomelidae). J Forest Sci. 2006; 52: 255–284.

[2] Yaman M Radek R *Nosema chaetocnemae* sp. n., a microsporidian (Microspora; Nosematidae) parasite of Chaetocnema tibialis (Chrysomelidae, Coleoptera). Acta Protozool. 2003; 42: 231–237.

[3] Yaman M Radek R Aslan I Ertürk Ö Characteristic features of *Nosema phyllotretae* Weiser 1961, a microsporidian parasite of *Phyllotreta atra* (Coleoptera: Chrysomelidae) in Turkey. Zool Stud. 2005; 44: 368–372.

[4] Yaman M Radek R Toguebaye B A new microsporidian of the genus *Nosema*, parasite of *Chaetocnema tibialis* (Coleoptera: Chrysomelidae) from Turkey. Acta Protozool. 2008; 47: 279–285.

[5] Yaman M Radek R Weiser J Toguebaye BS *Unikaryon phyllotretae* sp. n. (Protista, Microspora), a new microsporidian pathogen of Phyllotreta undulata (Coleoptera; Chrysomelidae). Eur J Protistol. 2010; 46(1):10–6. 19767185 10.1016/j.ejop.2009.07.001

[6] Yaman M Özcan N Radek R Linde A Lipa JJ Ultrastructure, characteristic features and occurrence of *Nosema leptinotarsae* Lipa 1968, a microsporidian pathogen of Leptinotarsa decemlineata (Coleoptera: Chrysomelidae). Acta Parasitol. 2011; 56: 1–7.

[7] Zhu F Shen Z Guo X Xu X Tao H Tang X Xu L A new isolate of *Nosema* sp. (Microsporidia, Nosematidae) from *Phyllobrotica armata* Baly (Coleoptera, Chrysomelidae) from China. J Invertebr Pathol. 2011; 106(2):339–42. 21035452 10.1016/j.jip.2010.10.005

[8] Toguebaye BS Marchand B Bouix G Microsporidia of Chrysomelidae. In: Petitpierre E Hsiao TH Jolivet PH editors. Biology of Chrysomelidae. Kluwer Academic Publishers, Boston, pp. 1988; 399–416.

[9] Sidor C Jodal I *Nosema melasomae* causing a disease of the poplar leaf beetle (Melasoma populi L., Chrysomelidae, Coleoptera). Zaštita Bilja 1986; 37: 243–249.

[10] Zeki H Toros S The effect of host on the adults of *Chrysomela populi*L. and Chrysomela tremulae F. (Col.: Chrysomelidae). Bitki Koruma Bülteni 1996; 36: 25–38.

[11] Yaman M Distribution and occurrence of the neogregarine pathogen, Ophryocystis anatoliensis (Apicomplexa) in populations of Chrysomela populi L. (Coleoptera: Chrysomelidae). Acta Protozool. 2017; 56: 283–288.

[12] Vávra J Larsson R Structure of Microsporidia. In: Wittner M editor. The Microsporidia and Microsporidiosis. ASM Press, Washington, 1999; pp. 7–84.

[13] Larsson JIR Identification of microsporidia. Acta Protozool. 1999; 38: 161–197.

[14] Yaman M Pınar Güngör F Gonca Güner B Radek R Linde A First report and spore ultrastructure of *Vairimorpha plodiae* (Opisthokonta: Microspora) from *Plodia interpunctella* (Lepidoptera: Pyralidae) in Turkey. Acta Parasitol. 2016; 61(2):228–31. 27078645 10.1515/ap-2016-0032

[15] Cheung WWK Wang JB Electron microscopic studies on *Nosema mesnili* Paillot (Microsporidia: Nosematidae) infecting the Malpighian tubules of *Pieris canidia* larva. Protoplasma 1995;186: 142–148.

[16] Toguebaye BS Marchand B *Nosema couilloudi* n.sp., Microsporidie parasite de *Nisotra* sp. (Coleoptera, Chrysomelidae): Cytopathologie et ultrastructure des stades de developpement. Protistologica, 1984; 20: 357–365.

[17] Toguebaye BS Marchand B Etude d’une infection microsporidienne due à *Nosema birgii* n.sp. (Microsporida, Nosematidae) chez *Mesoplatys cincta* Olivier, 1790 (Coleoptera, Chrysomelidae). Z Parasitenkd. 1986; 72: 723–737.

[18] Toguebaye BS Marchand B Observations en microscopie électronique à transmission des stades de développement de *Nosema nisotrae* sp.n. (Microsporida, Nosematidae) parasite de *Nisotra* sp. (Coleoptera, Chrysomelidae). Arch Protistenkd. 1989; 137: 69–80.

[19] Toguebaye BS Bouix G *Nosema galerucellae* sp.n., microsporidian (Protozoa, Microspora), parasite of *Galerucella luteola* Müller (Chrysomelidae, Coleoptera): Development cycle and ultrastructure. Eur J Protistol. 1989; 24: 346–353. 23195726 10.1016/S0932-4739(89)80005-7

[20] Weiser J Die Mikrosporidien als Parasiten der Insekten. Monogr Angew Entomol. 1961; 17: 1–149.

[21] Hostounský Z Weiser J *Nosema gastroideae* sp. n. (Nosematidae, Microsporidia) infecting *Gastrophysa polygoni* and *Leptinotarsa decemlineata* (Coleoptera: Chrysomelidae). Acta Entomol Bohemoslov. 1973; 70: 345–350.

[22] Hostounský Z Weiser J *Nosema polygrammae* sp. n. and *Plistophora fidelis* sp. n. (Microsporidia, Nosematidae) infecting *Polygramma undecimlineata* (Coleoptera: Chrysomelidae) in Cuba. - Vestn Cesk Spol Zool. 1975; 39: 104–110.

[23] Hostounský Z Weiser J A microsporidian infection in *Otiorrhynchus equestris* (Coleoptera, Curculionidae) in Cuba. Vestn Cesk Spol Zool. 1980; 44: 160–165.

[24] Lipa JJ *Nosema leptinotarsae* sp.n., a microsporidian parasite of the Colorado potato beetle, *Leptinotarsa decemlineata* (Say). J Inverteb. Pathol. 1968; 10: 111–115.

